# Mitral Valve Prolapse in Athletes: Prevalence, Arrhythmic Associations, and Clinical Implications—A Systematic Review

**DOI:** 10.3390/jcm14217475

**Published:** 2025-10-22

**Authors:** Andrea Sonaglioni, Gian Luigi Nicolosi, Michele Lombardo, Massimo Baravelli

**Affiliations:** 1 Division of Cardiology, IRCCS MultiMedica, 20123 Milan, Italy; michele.lombardo@multimedica.it (M.L.); massimo.baravelli@multimedica.it (M.B.); 2 Division of Cardiology, Policlinico San Giorgio, 33170 Pordenone, Italy; gianluigi.nicolosi@gmail.com

**Keywords:** athletes, mitral valve prolapse, prevalence, arrhythmias, pre-participation screening

## Abstract

**Background**: Mitral valve prolapse (MVP) is the most common valvular abnormality in the general population and has been linked to mitral regurgitation, arrhythmias, and sudden cardiac death. Its prevalence and prognostic significance in athletes remain uncertain, raising important questions for pre-participation screening, eligibility for competition, and long-term follow-up. **Methods**: We systematically searched PubMed, Scopus, and EMBASE databases from inception through August 2025 for original studies reporting MVP prevalence in athletes, diagnosed by echocardiography or pathological assessment. Data on study characteristics, diagnostic definitions, prevalence, arrhythmias, and outcomes were independently extracted by three reviewers. Methodological quality was appraised using the National Institutes of Health Quality Assessment Tool for Observational Cohort and Cross-Sectional Studies. **Results**: Twelve studies published between 1987 and 2024 met inclusion criteria, enrolling 19,463 athletes from diverse sports and competitive levels. A total of 407 MVP cases were identified, corresponding to a crude pooled prevalence of 2.4%. Prevalence estimates varied substantially (0.2–20%), reflecting heterogeneity in study populations and diagnostic definitions. When all studies were pooled using a random-effects model, the overall prevalence was 2.0% (95% CI 1.2–2.8%). A sensitivity analysis restricted to contemporary, unselected athletic cohorts yielded a prevalence of 1.1% (95% CI 0.4–1.9%), closely aligning with population-based estimates. Ventricular arrhythmias were more frequent than supraventricular arrhythmias, particularly in association with bileaflet prolapse, leaflet thickening, or significant mitral regurgitation. Most athletes were asymptomatic, and only one prospective study provided long-term follow-up, confirming a generally benign prognosis, though rare adverse events (atrial fibrillation, valve surgery) were documented. **Conclusions**: MVP is relatively uncommon in athletes and occurs at rates similar to the general population. In most cases, prognosis is favorable and should not preclude sports participation. Nonetheless, recognition of high-risk phenotypes with arrhythmogenic potential highlights the need for individualized evaluation and tailored surveillance strategies in sports cardiology practice.

## 1. Introduction

Mitral valve prolapse (MVP) is the most frequent valvular abnormality in developed countries, with a prevalence estimated at 2–3% in the general population [1]. It is defined echocardiographically as the systolic billowing of one or both mitral valve leaflets at least 2 mm beyond the plane of the mitral annulus into the left atrium, best visualized in the parasternal long-axis view [2,3]. Although most individuals with MVP experience a benign clinical course, the disorder has attracted enduring attention because of its potential complications, including mitral regurgitation (MR), infective endocarditis, atrial and ventricular arrhythmias, and, in selected cases, sudden cardiac death (SCD) [4,5,6,7,8]. In particular, a subgroup characterized by bileaflet or myxomatous prolapse, mitral annular disjunction (MAD), and complex ventricular arrhythmias (VAs) has recently been recognized as a distinct arrhythmogenic phenotype with heightened risk of adverse outcomes [9,10].

The relevance of MVP extends beyond the general population to athletic cohorts. Regular participation in competitive sports subjects the cardiovascular system to sustained hemodynamic load and adrenergic stimulation, which may amplify the arrhythmic substrate in susceptible individuals. Although MVP is often detected incidentally during pre-participation cardiovascular screening, its identification in an athlete carries important clinical and medico-legal implications, raising questions regarding eligibility for competition, need for further diagnostic testing, and intensity of follow-up [11,12]. Concern stems primarily from the potential contribution of MVP to exercise-related SCD, which remains a leading cause of mortality in young athletes [10,13].

Accurate assessment of MVP prevalence in athletes is therefore essential to inform clinical practice. However, estimates have historically been inconsistent, largely due to methodological heterogeneity. Earlier echocardiographic investigations, particularly those performed prior to 1999, frequently applied less specific diagnostic criteria based on M-mode or apical four-chamber imaging, leading to overestimation of prevalence [14,15,16,17,18,19,20,21]. More recent criteria [2,3], which account for the saddle-shaped nonplanarity of the mitral annulus [22,23], define MVP more strictly as leaflet displacement >2 mm beyond the annulus in the parasternal long-axis view. Adoption of these standards has reduced false positive diagnoses and led to substantially lower prevalence estimates. In a recent systematic review of echocardiographic studies in heterogeneous populations, the pooled prevalence of MVP decreased from 7.8% in studies conducted before 1999 to 2.2% in those performed thereafter [24].

Despite these advances, the true burden of MVP in athletes remains uncertain. Studies have reported variable rates across sports disciplines, competitive levels, and geographic regions. The lack of uniform diagnostic definitions, differences in study populations, and the absence of pooled analyses have limited the interpretability of available data. Moreover, while MVP is generally considered compatible with athletic participation, its association with VAs and SCD in selected cases underscores the need for precise epidemiological characterization in this setting [10,13,25].

For these reasons, a systematic review of the literature on MVP prevalence among athletes is warranted. By synthesizing evidence from echocardiographic studies performed in sporting populations and contextualizing them within the evolution of diagnostic criteria, the present work aims to provide a reliable estimate of MVP prevalence in athletes. This information is crucial for clinicians involved in pre-participation evaluation, for sports cardiology specialists assessing eligibility, and for researchers seeking to clarify the arrhythmogenic potential of MVP in athletic cohorts.

## 2. Materials and Methods

This systematic review was performed according to the PRISMA guidelines [26], and was registered in INPLASY database (INPLASY202590064).

### 2.1. Search Strategy

We conducted a comprehensive literature search to identify studies evaluating the prevalence of MVP among athletes. The search was performed in PubMed, Scopus, and EMBASE databases from inception through August 2025, without language restrictions. The search terms included combinations of “mitral valve prolapse,” “athletes,” “sports,” “echocardiography,” and “pre-participation screening.” Boolean operators and Medical Subject Headings (MeSH) were applied to maximize sensitivity. In addition, the reference lists of relevant systematic reviews and included studies were screened manually to identify additional eligible reports.

### 2.2. Eligibility Criteria

Studies were considered eligible if they met the following criteria: (1) original research published in peer-reviewed journals; (2) population of competitive or recreational athletes undergoing cardiovascular evaluation; (3) MVP diagnosis based on echocardiography or, in autopsy studies, pathological examination; and (4) report of MVP prevalence or raw numbers allowing calculation. No restriction was applied on athlete age, sex, type of sport, or competitive level. We excluded case reports, editorials, narrative reviews, abstracts without sufficient data, and studies in non-athletic populations. When duplicate publications from the same cohort were identified, the most comprehensive or recent report was included.

### 2.3. Study Selection and Data Extraction

Two investigators independently screened titles and abstracts retrieved from the search to exclude irrelevant records. Full texts were subsequently assessed for eligibility. Disagreements were resolved by consensus with a third reviewer. Data extraction was performed independently by three experienced cardiologists (A.S., M.B., and G.L.N.) in August 2025, using a standardized data collection form. For each study, the following information was collected: author, year of publication, country, population characteristics (type of sport, sample size, age, sex distribution), study design, diagnostic criteria for MVP, number and prevalence of MVP cases, and presence of arrhythmic complications, including VAs and supraventricular arrhythmias (SVAs). When available, follow-up information and clinical outcomes were also recorded. Extracted data were cross-checked for consistency and compiled in a summary table.

### 2.4. Risk of Bias Assessment

The methodological quality of the included studies was assessed using the National Institutes of Health (NIH) Quality Assessment Tool for Observational Cohort and Cross-Sectional Studies [27]. This instrument evaluates 14 domains, including clarity of the research question, population definition, participation rate, exposure and outcome measures, length of follow-up (if applicable), and adequacy of statistical analyses. Each study was independently evaluated by the three cardiologists who extracted the data, and judgments were categorized as good, fair, or poor quality according to NIH guidance.

### 2.5. Statistical Analysis

Prevalence estimates from each study were calculated as the number of MVP cases divided by the total sample size. Exact binomial 95% confidence intervals were computed using the Wilson score method. For pooled analyses, proportions were transformed using the Freeman–Tukey double arcsine method to stabilize variance. A random-effects model (DerSimonian–Laird) was applied to account for between-study heterogeneity. Both an overall pooled prevalence and a sensitivity analysis restricted to unselected, contemporary athlete cohorts were performed. Results are displayed as a forest plot, with individual study estimates and corresponding 95% confidence intervals, alongside pooled estimates. To assess potential publication bias, funnel plot inspection and Egger’s regression asymmetry test were performed. Funnel plot symmetry was visually evaluated, and statistical significance of small-study effects was determined using Egger’s test. Statistical analyses were conducted using Comprehensive Meta-Analysis version 3.0 (Biostat, Englewood, NJ, USA).

## 3. Results

### 3.1. Study Selection

**Figure 1:**
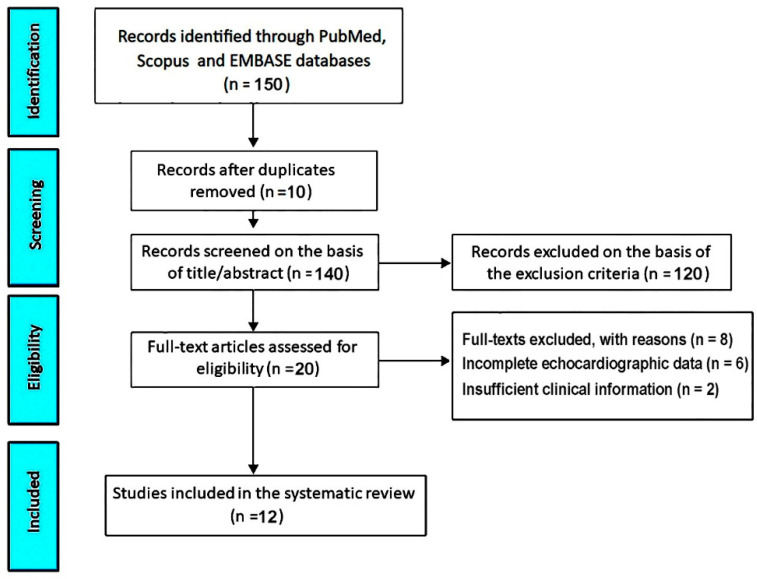
PRISMA flow diagram of study selection for the systematic review.

The initial database search retrieved 150 records. After removal of 10 duplicates, 140 titles and abstracts were screened. Of these, 120 records were excluded because they were clearly irrelevant, did not involve athletes, or did not evaluate MVP. Twenty full-text articles were assessed for eligibility. Among these, 8 studies were excluded: six due to incomplete or non-standardized echocardiographic data and two because of insufficient clinical information. Ultimately, 12 studies [28,29,30,31,32,33,34,35,36,37,38,39] met all inclusion criteria and were included in the present systematic review (Figure 1).

Clinical characteristics, diagnostic criteria, and main findings of the studies included in the systematic review are summarized in Table 1.

This systematic review included twelve studies published between 1987 and 2024, evaluating MVP prevalence in athletes and young sporting populations. Together, these studies enrolled a total of 19,463 participants, with the majority being male athletes, though the proportion of males varied across cohorts (range: 51–100%). Specifically, male representation was 71% in U.S. intercollegiate athletes [28], 83% in U.S. football players [29], 67% in young U.S. athletes undergoing echocardiography [31], 100% in Italian youth soccer players [32], 100% in elite African footballers screened in Switzerland/Gabon [33], 73% in Italian athletes with VAs [34], 76% in Italian paralympic athletes [35], 90% in Turkish multisport youth athletes [36], 100% in Canadian draft-eligible hockey players [37], 67% in a large Italian registry of competitive athletes [38], and 51% in the U.S. sudden cardiac death registry [39]. One large U.S. cohort (n = 2997) did not specify sex distribution [30].

The studies originated from multiple countries, including the USA (five studies), Italy (four studies), Switzerland (one study), Turkey (one study), and Canada (one study).

The mean age of athletes spanned a wide spectrum across studies. The youngest cohort was composed of pre-adolescent soccer players in Italy, with a mean age of 11 years [32], whereas the oldest were Italian paralympic athletes, with a mean age of 35 years [35]. Across the broader cohorts, mean ages ranged from 13 years in Turkish youth multisport athletes [36] to 30 years in a large Italian registry of competitive athletes [38]. Other studies reported mean ages in the late teens, including U.S. intercollegiate athletes (19.3 years) [28], American football players (19 years) [29], young U.S. athletes undergoing echocardiography (17.5 years) [31], elite Swiss footballers (18.6 years) [33], Canadian draft-eligible hockey players (18 years) [37], and U.S. sudden cardiac death cases (22 years) [39]. Some cohorts had unspecified ages [30].

### 3.2. Clinical Data

Overall, the pooled mean age across studies clustered in late adolescence and early adulthood (approximately 17–22 years), reflecting the predominant focus on screening competitive youth and young adult athletic cohorts. However, the inclusion of both very young pre-adolescents and older paralympic/elite athletes highlights the broad applicability of MVP screening across diverse athletic populations worldwide.

The pooled prevalence of MVP across all included studies was 2.4% (407 cases among 19,463 athletes). Substantial variation was observed across individual cohorts, with estimates ranging from as low as 0.2% in Canadian elite hockey players [37] to as high as 20% in an Italian referral cohort of athletes with VAs [34]. Excluding this highly selected group, most general athletic populations demonstrated prevalence rates between 0.3% and 3%, consistent with estimates in the general population.

Early North American studies [28,29] reported higher prevalence (11–15.6%), likely reflecting broader echocardiographic criteria and greater reliance on M-mode displacement. In contrast, more recent European cohorts employing standardized parasternal long-axis criteria [33,38] reported lower rates of 1–3%. These discrepancies highlight the impact of evolving diagnostic standards and differences in study populations.

When all studies were combined using a random-effects model, the overall pooled prevalence was 2.0% (95% CI 1.2–2.8%). This estimate was disproportionately influenced by older studies with permissive criteria and by arrhythmia-focused cohorts. A sensitivity analysis restricted to contemporary, unselected athletic populations [31,32,33,36,37,38] yielded a pooled prevalence of 1.1% (95% CI 0.4–1.9%), closely matching community-based estimates.

**Figure 2:**
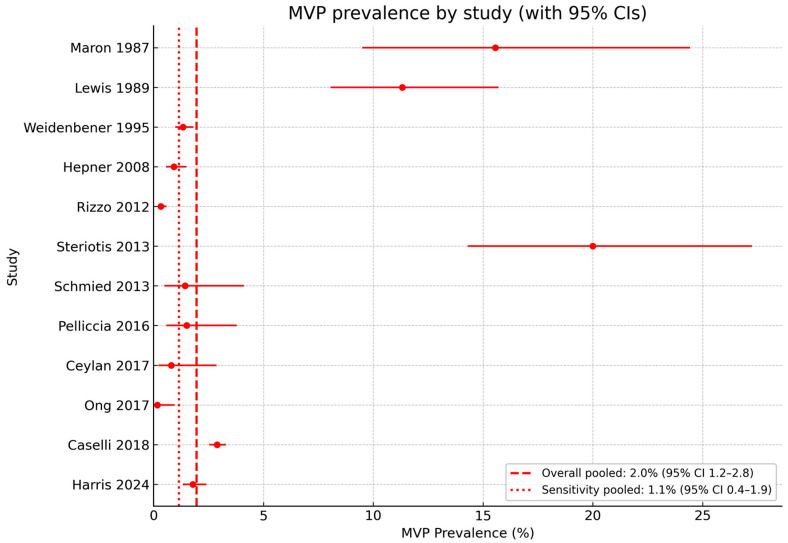
Forest plot of MVP prevalence in athletes across included studies [28,29,30,31,32,33,34,35,36,37,38,39], with overall and sensitivity pooled estimates (random-effects model). MVP, mitral valve prolapse.

A forest plot illustrating prevalence by study, along with pooled and sensitivity estimates, is presented in Figure 2.

From a clinical perspective, most athletes with MVP were asymptomatic at the time of assessment. Palpitations, exertional intolerance, or chest discomfort were occasionally reported but were generally nonspecific and not predictive of structural abnormalities. Physical examination yielded variable findings: systolic clicks or murmurs were sometimes present but lacked sufficient sensitivity or specificity to guide diagnosis, underscoring the superiority of echocardiography in confirming MVP. Importantly, the Harris registry [39] provided a contrasting autopsy-based perspective, identifying MVP as an under-recognized cause of sudden cardiac death in young athletes. Pathological features associated with these fatal cases included bileaflet prolapse, myxomatous leaflet degeneration, and localized myocardial fibrosis, particularly of the posterolateral left ventricular wall. These findings highlight that while MVP is generally a benign incidental finding in athletes, a small subset with high-risk morphologic variants may carry an arrhythmogenic potential that remains clinically silent during life.

### 3.3. ECG and Arrhythmia Data

Electrocardiographic data and arrhythmia outcomes were variably reported, with greater emphasis on VAs than SVAs.

Ventricular arrhythmias were most comprehensively assessed in two studies. Steriotis et al. [34] reported that MVP was present in 20% of athletes referred specifically for VAs, confirming a potential causal association. Caselli et al. [38] identified VAs in 0.8% of athletes with MVP in their large competitive cohort, often in association with MR or MAD. Other studies noted isolated cases of VAs, including Pelliccia et al. [35] in paralympic athletes and Ceylan et al. [36] in pediatric athletes. Schmied et al. [33], in contrast, reported no VAs among elite African footballers with MVP.

Supraventricular arrhythmias were less common but still observed. Caselli et al. [38] reported one case of atrial fibrillation requiring hospitalization during long-term follow-up. Ceylan et al. [36] detected a single case of supraventricular tachycardia by Holter monitoring in a pediatric athlete. Pelliccia et al. [35] documented four cases of SVAs in paralympic athletes, corresponding to 1.5% of that cohort. All other studies did not report SVAs, indicating that these events were sporadic and uncommon.

Overall, arrhythmia prevalence was low in unselected athletic populations, but certain subgroups—particularly those with bileaflet prolapse, MR, or MAD—appeared more vulnerable to rhythm disturbances. The Harris registry [39] confirmed the arrhythmogenic potential of MVP, documenting frequent myocardial fibrosis in autopsied sudden death cases, most often in the posterolateral left ventricle.

### 3.4. Echocardiographic Data

Echocardiography emerged as the cornerstone diagnostic modality in virtually all prospective investigations, given its non-invasive nature, widespread availability, and capacity to provide direct visualization of mitral valve morphology and motion. The prevailing diagnostic definition was systolic displacement of one or both mitral leaflets ≥2 mm beyond the annular plane in the parasternal long-axis view, consistent with modern echocardiographic standards [32,35,38]. Earlier investigations, particularly those from the 1980s, frequently incorporated M-mode criteria, with prolapse defined as posterior displacement ≥3 mm [28,29]. This methodological difference likely accounts for the higher prevalence estimates reported in those cohorts, as M-mode has a greater propensity to overestimate leaflet excursion. In contrast, Schmied et al. [33] applied more stringent echocardiographic thresholds, requiring both systolic displacement and leaflet thickening >5 mm, which reduced the observed prevalence to 1.4%.

Across the pooled populations, MVP identified by echocardiography was generally mild in severity and not associated with clinically significant mitral regurgitation in most athletes [31,34,38]. Morphologic variants such as leaflet thickening, redundancy, or MAD were described in selected cohorts but not consistently quantified [37,39]. The clinical relevance of these features remains debated, although accumulating evidence suggests they may contribute to arrhythmic risk in certain subgroups [39]. Importantly, echocardiography frequently identified MVP in athletes with normal physical examination and surface ECG findings [32,36], reinforcing its utility as a sensitive diagnostic modality within preparticipation cardiovascular screening programs. Collectively, these findings highlight that while MVP in athletes is typically benign and of limited hemodynamic consequence, its detection relies almost exclusively on imaging, underscoring echocardiography’s central role in both epidemiologic surveillance and individual risk stratification.

### 3.5. Follow-Up Data

Among all the studies included, only Caselli et al. [38] provided systematic long-term follow-up. Over a mean of 8 ± 2 years, the prognosis of MVP in athletes was generally benign. No cases of sudden cardiac death occurred, though a small subset required mitral valve surgery for progressive regurgitation, and one athlete developed atrial fibrillation necessitating hospitalization. Risk stratification identified arrhythmias, MR, and MAD as predictors of adverse outcomes.

The Harris registry [39] added complementary retrospective evidence, revealing that MVP may underlie 1.8% of sudden cardiac deaths in young athletes. Pathological analysis highlighted bileaflet prolapse and ventricular fibrosis as potential substrates for malignant arrhythmias. While not a prospective follow-up study, these findings underscore the importance of recognizing high-risk MVP phenotypes.

### 3.6. Risk of Bias Assessment

The 12 included studies were evaluated using the NIH Quality Assessment Tool for Observational Cohort and Cross-Sectional Studies. The Cohen’s Kappa coefficient for the agreement between the reviewers in the RoB assessment indicated substantial agreement, κ = 0.81. Eleven studies scored 5/14 and were rated fair quality, while one [38] scored 8/14 and was judged good quality (Table 2).

**Figure 3:**
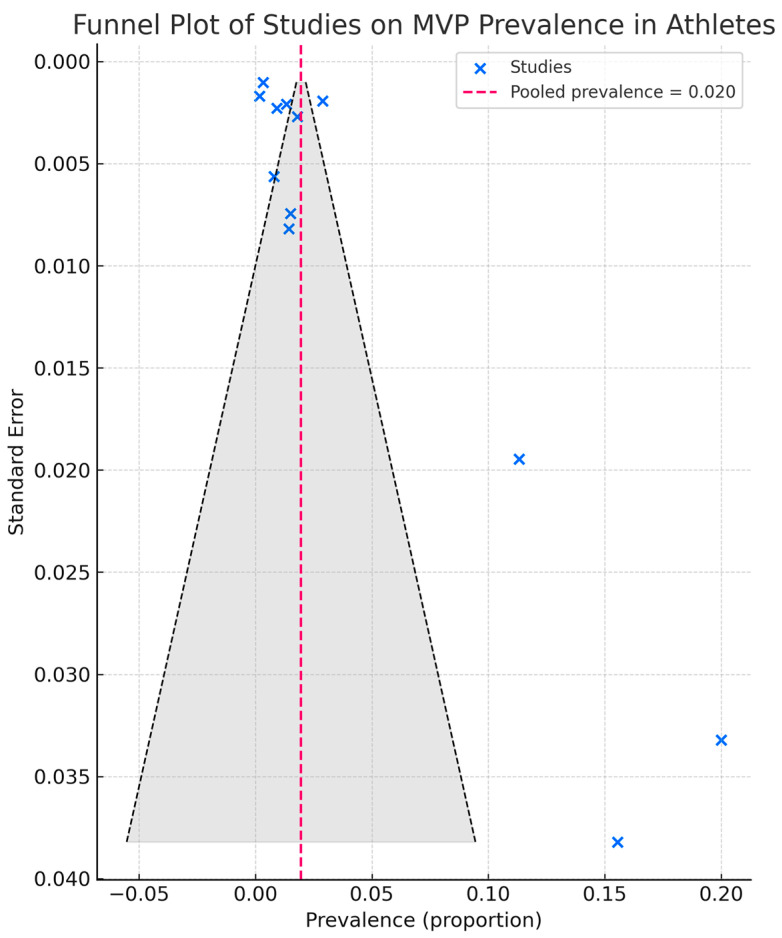
Funnel plot of studies included in the systematic review of MVP prevalence in athletes. Each blue dot represents an individual study estimate plotted against its standard error. The vertical red dashed line indicates the mean prevalence across studies. The gray dashed lines and shaded area represent the pseudo–95% confidence limits. MVP, mitral valve prolapse.

All studies clearly reported their objectives and defined their populations. However, participation rates, sample size calculations, blinding, and adjustment for confounding were not described. Most investigations were cross-sectional pre-participation screenings, lacking temporal sequence or repeated assessments, with the exception of Caselli et al. [38], which included longitudinal follow-up. Outcome measures—MVP diagnosis by echocardiography—were consistently well defined, supporting internal validity. Assessment of publication bias using funnel plot inspection (Figure 3) and Egger’s regression test (*p* = 0.74) did not reveal significant evidence of small-study effects. However, given the limited number of included studies (n = 12), the statistical power of these analyses remains low.

## 4. Discussion

### 4.1. Interpretation of the Main Findings

This systematic review confirms that MVP is uncommon in athletes and occurs at rates comparable to the general population. However, the substantial variability in reported prevalence underscores the lack of standardized diagnostic definitions and the methodological heterogeneity of earlier studies. Differences in echocardiographic criteria, imaging quality, and population selection explain the wide range of prevalence estimates across decades.

Rather than reaffirming known prevalence figures, the present synthesis emphasizes the need to interpret historical data within the evolving diagnostic landscape. Studies applying M-mode or apical four-chamber criteria frequently overestimated leaflet excursion, whereas modern parasternal long-axis views yield more reliable detection. This diagnostic evolution is central to understanding the apparent decline in prevalence over time and represents a crucial methodological gap for future research.

Arrhythmic manifestations were infrequent in unselected cohorts but concentrated among athletes with bileaflet prolapse, MAD, or significant regurgitation. These findings support the emerging concept of an “arrhythmogenic MVP” phenotype, in which structural and myocardial alterations may predispose to ventricular arrhythmias. Importantly, most athletes without these high-risk markers remain clinically stable, reinforcing the overall benign prognosis of MVP in this population.

### 4.2. Evidence Gaps and Quality Considerations

The limited number of longitudinal studies and the predominance of cross-sectional screening designs restrict firm conclusions about the natural history of MVP in athletes. Only one cohort provided systematic follow-up, confirming the generally favorable outcome but highlighting the scarcity of prospective data on progression, arrhythmia burden, and long-term performance. Moreover, female athletes were markedly underrepresented, preventing sex-specific analyses despite evidence that MVP morphology and arrhythmic risk may differ by sex.

The methodological quality of available studies was modest, with few employing blinded assessment or adjustment for confounders. These weaknesses reduce the certainty of pooled estimates and call for multicenter prospective investigations with standardized echocardiographic and CMR criteria to improve reproducibility and external validity.

### 4.3. Comparison Between MVP Prevalence in Athletes and in the General Population

The prevalence of MVP in athletes does not substantially differ from that reported in unselected cohorts. Large-scale epidemiological studies using modern echocardiographic criteria show a MVP prevalence of approximately 2–3% in community samples [1,2], whereas earlier studies relying on M-mode reported much higher rates, up to 5–7% or more [14,15,16,17,18,19,20,21]. Our systematic review aligns with these observations, as athletic cohorts evaluated after 1999 with stricter criteria demonstrated prevalence estimates within the same range.

Recent reviews confirm that MVP is generally benign, with only a minority progressing to significant MR, arrhythmias, or adverse outcomes [40,41], consistent with findings in athletes. The potential arrhythmic burden deserves particular consideration: population studies indicate that VAs are observed in 20–30% of MVP cases [42], while supraventricular arrhythmias—mainly atrial fibrillation—are linked to long-standing MR or left atrial dilation [43,44]. In athletes, the distribution is similar, with VAs more frequent than SVAs and associated with bileaflet prolapse, MAD, or myocardial fibrosis [45]. Recent multimodality imaging studies further emphasize that excessive leaflet motion, systolic curling, inferolateral wall stress, and fibrosis detectable by CMR are associated with higher arrhythmic risk [46,47]. Consequently, current evidence does not suggest that athletes with MVP carry a higher intrinsic arrhythmic risk than non-athletes, though careful surveillance is warranted in subgroups with high-risk morphologic or functional features [10,13,48,49].

### 4.4. Clinical Implications for Screening, Eligibility, and Surveillance in Athletes with MVP

The detection of MVP in athletes has important clinical implications for screening, eligibility, and surveillance. Pre-participation cardiovascular evaluation remains a cornerstone of SCD prevention [50]. While most guidelines recommend history and physical examination as the initial tools, echocardiography may be considered in selected individuals with abnormal auscultation, symptoms, or family history of sudden death [11]. Identification of MVP in such contexts should not lead to automatic disqualification. Rather, management should be individualized, integrating MR severity, arrhythmic profile, and structural risk factors such as bileaflet prolapse or MAD [51].

From an eligibility perspective, international consensus statements emphasize that athletes with mild MVP and no evidence of significant regurgitation or arrhythmias can participate in all competitive sports [12]. Restrictions apply to those with high-risk features, including moderate-to-severe MR, LV dysfunction, syncope of arrhythmic origin, or complex VAs during exercise testing [52,53]. Comprehensive evaluation should extend beyond echocardiography to include ambulatory ECG and exercise testing. In selected cases, CMR can refine risk stratification by detecting fibrosis of the inferolateral ventricular wall or papillary muscles [40,54,55].

Follow-up frequency should be proportionate to individual risk: athletes with uncomplicated MVP require reassessment every few years, while those with regurgitation, arrhythmias, or structural abnormalities necessitate closer monitoring [56]. The recognition of arrhythmogenic MVP reinforces the importance of vigilance even in asymptomatic athletes, since adrenergic stimulation inherent to intense exercise can amplify electrical instability. Shared decision-making among athletes, families, and sports medicine teams remains essential to balancing the low absolute risk of adverse events against the physical and psychosocial benefits of competitive sport.

Beyond clinical management, this synthesis underscores critical evidence gaps that affect real-world decision-making. The absence of standardized diagnostic definitions hinders the consistent identification of high-risk morphologic variants. Likewise, the paucity of longitudinal outcome data and the marked underrepresentation of female athletes limit the understanding of sex-specific risk and progression. Addressing these deficiencies in future multicenter, prospective studies will be essential to advance beyond reliance on guideline extrapolation and toward truly evidence-based athletic cardiology.

### 4.5. Chest Shape Assessment in Athletes

In recent years, our research group developed the modified Haller index (MHI), a novel, noninvasive anthropometric measure of chest shape conformation [57]. The MHI is calculated by dividing the latero-lateral external thoracic diameter—measured with a rigid ruler and level at the lower third of the sternum—by the antero-posterior (A-P) internal thoracic diameter, obtained from the echocardiographic parasternal long-axis view as the distance between the true apex of the sector and the posterior wall of the descending aorta located just behind the left atrium.

In previous investigations, we demonstrated that MVP individuals frequently exhibit a concave chest wall or varying degrees of anterior sternal depression/pectus excavatum (PE) [58]. These anatomical characteristics were often associated with benign functional profiles, including mild reductions in basal longitudinal strain on speckle-tracking echocardiography [59], trivial MR during exercise stress testing, and favorable mid- to long-term outcomes [60]. Notably, these findings predominated among subjects with an MHI >2.5 and/or an A-P thoracic diameter ≤13.5 cm.

**Figure 4:**
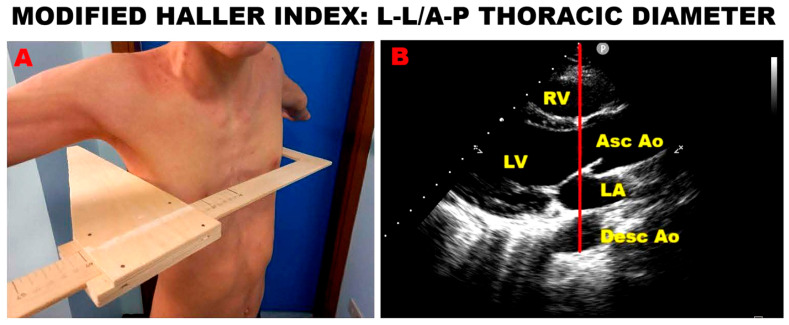
Evaluation of the modified Haller index in a young athlete with pectus excavatum. (**A**) Measurement of the latero-lateral external thoracic diameter using a rigid ruler and level positioned at the lower third of the sternum. (**B**) Measurement of the antero-posterior internal thoracic diameter (red line) obtained in the parasternal long-axis echocardiographic view, defined as the distance between the sector apex and the posterior wall of the descending aorta located immediately behind the left atrium. Ao, aorta, Asc, ascending; Desc, descending; LA, left atrium; LV, left ventricle; RV, right ventricle.

An example of MHI assessment in a young athlete with PE is shown in Figure 4.

Chest wall conformation, particularly the A-P thoracic diameter, has been increasingly recognized as an important determinant of cardiovascular findings in athletes. A narrow A-P diameter (≤13.5 cm) or concave thoracic shape has been shown to predispose to MVP, most often associated with only trivial MR and benign forms of MAD [61]. According to the “mechanical theory,” extrinsic sternal compression and altered chest geometry exert abnormal traction on the submitral apparatus, promoting leaflet billowing and subtle annular disjunction without causing major myocardial injury.

Athletes with this thoracic phenotype often display false-positive findings during stress echocardiography, such as transient wall motion abnormalities unrelated to coronary disease. These apparent anomalies likely result from dynamic ventricular dyssynchrony or paradoxical septal motion secondary to thoracic compression rather than ischemia [62]. Incorporating chest morphology assessment—via the MHI or direct echocardiographic A–P measurement—into pre-participation evaluations may therefore assist clinicians in distinguishing benign anatomic variants from clinically significant MVP. Such integration would help identify low-risk athletes who can safely continue sports activity, reduce unnecessary disqualifications, and optimize resource allocation for higher-risk individuals requiring closer surveillance.

### 4.6. Limitations of the Included Studies

Several limitations of the included studies should be acknowledged. First, substantial heterogeneity existed among the 12 investigations regarding population characteristics, competitive level, and type of sport, complicating both direct comparisons and the aggregation of prevalence estimates. Most cohorts comprised young male soccer players or mixed athletic groups from Europe and North America, leaving important subgroups—such as female athletes, endurance sports participants, and individuals from non-Western regions—underrepresented. Second, diagnostic definitions of MVP varied considerably. Earlier studies often relied on M-mode or apical four-chamber displacement, techniques now recognized to overestimate prevalence, whereas more recent work applied standardized parasternal long-axis criteria. The coexistence of these differing approaches across the literature introduces variability and limits comparability. Third, three investigations employed a retrospective design, increasing susceptibility to selection and information bias, particularly in the evaluation of arrhythmic outcomes and follow-up data. Fourth, longitudinal data were largely lacking; only one study systematically reassessed athletes over time, restricting insight into disease progression and prognosis. Finally, the overall methodological quality of the evidence was modest. According to the NIH Quality Assessment Tool, 11 of the 12 studies were rated as fair and only one as good. Common methodological weaknesses included retrospective or cross-sectional designs, limited follow-up, absence of blinding or confounder adjustment, and incomplete reporting of participation rates.

## 5. Conclusions

This systematic review of 12 studies including nearly 20,000 athletes shows that MVP is uncommon but consistently present. Across all cohorts, the pooled prevalence was 2.4%; a random-effects meta-analysis yielded 2.0% (95% CI 1.2–2.8%), driven by older permissive definitions and referral cohorts. Restricting analyses to contemporary, unselected athletes, prevalence was 1.1% (95% CI 0.4–1.9%), similar to the general population. Most cases were mild and hemodynamically insignificant, supporting continued sports participation in the majority. Arrhythmic events were infrequent but clustered in subgroups with high-risk phenotypes such as bileaflet prolapse, leaflet redundancy, or significant regurgitation. Overall, these findings underscore the generally favorable prognosis of MVP in athletes while emphasizing the need for individualized surveillance. Assessment of chest wall morphology using the MHI may help clinicians identify low-risk athletes and differentiate benign structural variants from clinically significant phenotypes. Future prospective, standardized, and sex-balanced studies with extended follow-up are essential to clarify the natural history of MVP in athletes and to refine evidence-based risk stratification.

## Figures and Tables

**Table 1:** Clinical characteristics, diagnostic criteria, and main findings of the included studies [28,29,30,31,32,33,34,35,36,37,38,39]. LA, left atrium; MVP, mitral valve prolapse; NR, not reported, NS, not specified; P, prospective; R, retrospective; SCD, sudden cardiac death; SVA, supraventricular arrhythmias; VAs, ventricular arrhythmias.

Author (Year)	Population (Size)	Av. Age (yrs) (% Males)	Study Design	MVP Diagnostic Criteria	MVP Cases (%)	VAs (%)	SVAs (%)	Main Findings
Maron B.J. et al. USA (1987) [28]	Intercollegiate Athletes (90)	19.3 (71)	P	M-mode: ≥3 mm late systolic or pansystolic displacement of one/both mitral leaflets posterior to line of closure. 2D echo: displacement of mitral leaflets beyond annular plane into LA in long-axis view or apical 4-chambers views.	14 (15.6)	1 (1.1)	NR	Echocardiography improves MVP detection; auscultation alone often misleading.
Lewis J.F. et al. USA (1989) [29]	Preparticipation echo screening (football) (265)	19 (83)	P	M-mode: ≥3 mm late systolic or pansystolic displacement of one/both mitral leaflets posterior to line of closure. 2D echo: displacement of mitral leaflets beyond annular plane into LA in long-axis view or apical 4-chambers views.	30 (11)	NR	NR	Feasibility of large-scale echo screening; MVP generally benign in athletes.
Weidenbener E.J. et al. USA (1995) [30]	Athletic (2997)	NS	R	Parasternal long- and short-axis views chosen as screening views	40 (1.3)	NR	NR	Recommended adding echocardiography to pre-participation exam despite cost concerns.
Hepner A.D. et al. USA (2008) [31]	Young athletes (1742)	17.5 (67.3)	R	Use of multiple echocardiographic cross sections (rather than the long-axis view alone)	16 (0.9)	NR	NR	MVP in young athletes usually asymptomatic with favorable short-term outcome.
Rizzo M. et al. Italy (2012) [32]	Youth soccer players (3100)	11 (100)	P	Single or bileaflet systolic protrusion ≥2 mm beyond the long-axis annular plane into LA	10 (0.32)	NR	NR	Echocardiography detected MVP cases undiagnosed by ECG or clinical exam.
Schmied C. et al. Switzerland (2013) [33]	FIFA elite footballers (210)	18.6 (100)	P	Leaflet thickening >5 mm + systolic displacement >2 mm in parasternal long-axis view	3 (1.4)	0(0.0)	0 (0.0)	Applied strict criteria; identified MVP in African athletes without major complications.
Steriotis A.K. et al. Italy (2013) [34]	Young athletes with ventricular arrhythmias (145)	17.3 (73)	P	Single or bileaflet systolic protrusion ≥2 mm beyond the long-axis annular plane into LA	29 (20)	145 (100)	NR	Found MVP associated with ventricular arrhythmias in competitive athletes.
Pelliccia A. et al. Italy (2016) [35]	Paralympic athletes (267)	35 (76)	P	Single or bileaflet systolic protrusion ≥2 mm beyond the long-axis annular plane into LA	4 (1.5)	5 (1.9)	4 (1.5)	MVP was rare; overall cardiac abnormalities infrequent in Paralympic athletes.
Ceylan Ö et al. Turkey (2017) [36]	Youth athletes (football, basketball, volleyball, swimming) (250)	13 (89.6)	P	Single or bileaflet systolic protrusion ≥2 mm beyond the long-axis annular plane into LA	2 (0.8)	1 (0.4)	1 (0.4)	Pediatric cardiology screening revealed MVP and other conditions requiring restrictions.
Ong G. et al. Canada (2017) [37]	Draft-eligible elite hockey players (592)	18 (NS)	P	Single or bileaflet systolic protrusion ≥2 mm beyond the long-axis annular plane into LA	1 (0.2)	NR	NR	Echocardiography in elite hockey players occasionally revealed benign MVP.
Caselli S. et al. Italy (2018) [38]	Competitive athletes (7449)	30 (67)	P	Single or bileaflet systolic protrusion ≥2 mm beyond the long-axis annular plane into LA	215 (2.9)	62 (0.8)	1 (0.01)	MVP prognosis usually benign; adverse outcomes linked to arrhythmias or regurgitation.
Harris K.M. et al. USA (2024) [39]	US autopsy SCD registry (2406)	22 (51)	R	Pathologic criteria: bileaflet myxomatous involvement, chordal thickening/elongation, interstitial or replacement LV fibrosis	43 (1.8)	NR	NR	Autopsies revealed arrhythmogenic MVP with fibrosis as cause of sudden death.

**Table 2:** Quality Assessment Tool for Observational Cohort and Cross-Sectional Studies [28,29,30,31,32,33,34,35,36,37,38,39]. Q1–Q14 items are accessible from the following URL: https://www.nhlbi.nih.gov/health-topics/study-quality-assessment-tools (accessed on 25 August 2025).

Study Name	Q1	Q2	Q3	Q4	Q5	Q6	Q7	Q8	Q9	Q10	Q11	Q12	Q13	Q14	Quality (Total Quality Score)
Maron B.J. et al. (1987) [28]	Yes	Yes	NR	Yes	No	No	No	No	Yes	No	Yes	NR	NR	No	5
Lewis J.F. et al. (1989) [29]	Yes	Yes	NR	Yes	No	No	No	No	Yes	No	Yes	NR	NR	No	5
Weidenbener E.J. et al. (1995) [30]	Yes	Yes	NR	Yes	No	No	No	No	Yes	No	Yes	NR	NR	No	5
Hepner A.D. et al. (2008) [31]	Yes	Yes	NR	Yes	No	No	No	No	Yes	No	Yes	NR	NR	No	5
Rizzo M. et al. (2012) [32]	Yes	Yes	NR	Yes	No	No	No	No	Yes	No	Yes	NR	NR	No	5
Schmied C. et al. (2013) [33]	Yes	Yes	NR	Yes	No	No	No	No	Yes	No	Yes	NR	NR	No	5
Steriotis A.K. et al. (2013) [34]	Yes	Yes	NR	Yes	No	No	No	No	Yes	No	Yes	NR	NR	No	5
Pelliccia A. et al. (2016) [35]	Yes	Yes	NR	Yes	No	No	No	No	Yes	No	Yes	NR	NR	No	5
Ceylan Ö. et al. (2017) [36]	Yes	Yes	NR	Yes	No	No	No	No	Yes	No	Yes	NR	NR	No	5
Ong G. et al. (2017) [37]	Yes	Yes	NR	Yes	No	No	No	No	Yes	No	Yes	NR	NR	No	5
Caselli S. et al. (2018) [38]	Yes	Yes	NR	Yes	No	Yes	Yes	No	Yes	No	Yes	NR	Yes	No	8
Harris K.M. et al. (2024) [39]	Yes	Yes	NR	Yes	No	No	NR	No	Yes	No	Yes	NR	NR	No	5

## Data Availability

Data extracted from included studies will be publicly available on Zenodo (https://zenodo.org).
